# Draft Genome Sequence of *Bacillus thuringiensis* Strain DAR 81934, Which Exhibits Molluscicidal Activity

**DOI:** 10.1128/genomeA.00175-12

**Published:** 2013-03-21

**Authors:** Aisuo Wang, Julie Pattemore, Gavin Ash, Angela Williams, James Hane

**Affiliations:** a School of Agricultural and Wine Sciences, Charles Sturt University, Wagga Wagga, NSW, Australia; b EH Graham Centre for Agricultural Innovation, Charles Sturt University, Wagga Wagga, NSW, Australia; c Black Box Informatics, Perth, WA, Australia

## Abstract

*Bacillus thuringiensis* has been widely used as a biopesticide for a long time. Its molluscicidal activity, however, is rarely realized. Here, we report the genome sequence of *B. thuringiensis* strain DAR 81934, a strain with molluscicidal activity against the pest snail *Cernuella virgata*.

## GENOME ANNOUNCEMENT

*Bacillus thuringiensis* is widely used as a biopesticide due to its capability to produce insecticidal crystal proteins (ICPs) (1). However, a new *B. thuringiensis* strain, DAR 81934, isolated in our laboratory, demonstrated molluscicidal activity toward the pest snail *Cernuella virgata* (data not shown), which confirms the findings of other researchers (2, 3). To further study the genetic basis of this *B. thuringiensis* strain, we sequenced its whole genome. The strain has been lodged in the Plant Pathology Herbarium (DAR) culture collection, Orange, NSW, Australia (strain DAR 81934).

The genome of *B. thuringiensis* DAR 81934 (Bt 81934) was sequenced at the Australian Genome Research Facility (AGRF) using an Illumina HiSeq 2000 instrument. The total novel isolate reads were aligned to three reference genomes (those of *B. thuringiensis* serovar Konkukian strain 97-27, *B. thuringiensis* strain Al Hakam, and *B. thuringiensis* BMB171) via the Burrows-Wheeler Aligner (BWA) (parameters: -o3 -e3 -d5 -i5 -R50) (4). Local realignment around indels was performed with the Genome Analysis Toolkit (GATK) v1.5.20 (5). Regions of >50× coverage were used as the basis for contigs. Contigs were first scaffolded via read-pairing relationships with SSPACE 2.0 (6) and then scaffolded via Optimal Syntenic Layout of unfinished assemblies (OSLay) (7). Scaffolding gaps were closed using the Beijing Genomics Institute (BGI) GapCloser 1.2. Novel isolate reads that were not aligned to the three reference genomes were *de novo* assembled with Velvet 1.2 using a kmer length of 41 bp (8). All the assemblies were combined via HaploMerger (9). As haplotype merging can potentially introduce single nucleotide polymorphism (SNP)-like assembly errors at merged sites (10), raw reads were back-aligned to the final assembly and the sequence consensus was confirmed via GATK (5).

The 5.94-Mb genome of Bt 81934 contains two components: a 5.69-Mb chromosome sequence (scaffolds 1 to 9), and a 0.25-Mb plasmid sequence (scaffolds 10 to 11). The average G+C content of the chromosome sequence is 33.67%, while that of the plasmid sequence is 32.75%. Protein-coding genes were predicted *in silico* via GeneMark-S (11), producing 6,042 genes, with 5,797 genes in the chromosome and 248 genes in the plasmid. tRNA and rRNA genes were identified by tRNAscan-SE (12) and RNAmmer (13), respectively. The whole genome contains 73 tRNA genes and 24 rRNA genes (all in scaffold 1). Bt toxin genes were predicted via BtToxinScanner (14). Two *cry* genes were identified in scaffold 10. Additionally, the BtToxin_Scanner database of *cry*, *cyt*, and *vip* genes was compared to the predicted protein dataset and scaffold sequences via BLASTp and tBLASTn, respectively. This produced matches to a further four toxin candidate genes in scaffolds 1 and 3.

In summary, this is the first report for the genome sequence of a *B. thuringiensis* strain with molluscicidal activity. The genome data indicate that Bt 81934 harbors *cry* and *vip* genes not only in the plasmid sequence, but also in the chromosome sequence. The availability of the genome data will facilitate the understanding of Bt endotoxin protein production and the genetic basis of its molluscicidal activity.

### Nucleotide sequence accession numbers. 

The draft genome sequence for Bt 81934 has been included in the GenBank Whole-Genome Shotgun (WGS) database under the accession no. ANPK01000001 to ANPK01000083.
